# Incident portal vein thrombosis in liver transplant recipients in New Zealand: Predictors of risk and validation of portal vein thrombosis risk index calculator

**DOI:** 10.3389/frtra.2022.1042684

**Published:** 2022-12-05

**Authors:** Paras Garg, Barry Harrison, Edward J. Gane

**Affiliations:** ^1^New Zealand Liver Transplant Unit, Auckland City Hospital, Auckland, New Zealand; ^2^Faculty of Medicine, University of Auckland, Auckland, New Zealand

**Keywords:** portal vein thrombosis, liver transplantation, risk prediction models, waiting list, NASH

## Abstract

The risk of spontaneous portal vein thrombosis (PVT) is increased in patients on the waiting list for liver transplantation and increases perioperative risks. A predictive PVT risk-index (PVT-RI) calculator has been proposed to determine the risk of incident PVT. We performed a retrospective analysis on adult liver transplant recipients at the NZ Liver Transplant Unit between January 1998 and February 2020. Variables reviewed included age at listing and transplantation, wait time from listing to transplant, indication for listing, gender, ethnicity, etiology of liver disease, listing MELD score, hepatocellular carcinoma (HCC), moderate-to-severe ascites, hepatic encephalopathy (>grade 2), transjugular intrahepatic portosystemic shunt (TIPSS), spontaneous bacterial peritonitis (SBP), and diabetes. Incident PVT was determined by imaging of patients while on the waiting list and assessment at transplantation. A total of 553 out of 706 patients met the inclusion criteria. Of those 553, 18 (3.3%) patients had incident PVT. The PVT-RI calculator was not validated in our cohort with only one of those 18 (6%) patients having a score of >4.6 (high risk cut-off score). Longer waiting time for transplant and listing for liver failure rather than HCC were independent predictors of the risk of incident PVT. There was no statistically significant difference in the incidence of PVT in viral vs. non-viral and cholestatic vs. non-cholestatic etiology of chronic liver disease. Patients with longer waiting times on the transplant waiting list should be monitored regularly for PVT.

## Introduction

The hepatic hemostasis environment in cirrhotic patients is a difficult balance because of a relative deficiency of both procoagulant and anticoagulant factors. This balance can be easily tipped between bleeding and thrombosis, depending on the provocation (1–3). Portal vein thrombosis is a well-recognized complication in cirrhotic patients awaiting liver transplant (4). It is no longer considered a contraindication to liver transplantation with advanced imaging modalities and expertise in liver transplantation techniques including thrombectomy or insertion of an extra-anatomical mesenteric graft (5). However, patient outcomes remain inferior in candidates with PVT in both the pretransplant and posttransplant time periods. Liver transplant recipients with PVT have a lower health-related quality of life, a higher rate of hepatic decompensation, and decreased post-transplant survival (6–9). Baseline factors associated with an increased incidence of PVT include reduced portal vein blood flow, more severe liver disease, presence of HCC, and underlying liver disease other than chronic viral hepatitis etiology (10–14). Other contributing factors are acquired hypercoagulability through elevated levels of prohemostatic von Willebrand factor (vWF) and decreased levels of the naturally occurring anticoagulants protein C, protein S, and antithrombin as well as heparin cofactor II (15). Rates of the Janus kinase 2 V6i7F mutation, the antiphospholipid antibody syndrome, the inherited factor V Leiden, prothrombin G20210A, and methylenetetrahydrofolate reductase C677T mutations are also more prevalent in cirrhosis patients with PVT (16, 17).

A predictive PVT risk index scoring has been proposed to determine the risk of incident PVT in patients listed for liver transplantation (18).

PVT-RI = 0.335^*^NASH + 0.095^*^MELD score + 0.126^*^moderate/severe ascites + 0.028^*^age – 0.261^*^African American race.

We aimed to review patient characteristics in our cohort of adult (age ≥ 18) liver transplant recipients in New Zealand at the time of listing and apply the PVT-RI calculator to ascertain if it can be validated in our population with end-stage liver disease and cirrhosis.

## Patients and methods

We performed a retrospective analysis of all adults who underwent liver transplantation at the NZ liver transplant unit between January 1998 and February 2020. Pre and post-transplant data were retrieved from the New Zealand Liver Transplant Unit national database and review of individual patient clinical records, both online and hard copy notes. This database has been maintained ever since the beginning of the liver transplantation program in NZ in 1998. It contains all the relevant information about the patients, etiologies of their chronic liver disease, features of their disease and complications at the time of listing, waiting time from listing to transplant, etc. It is saved and updated regularly as a soft copy version in a secure location in our unit's protected hard drive. We have approval from the New Zealand ethics committee for this database.

Patients who underwent either emergency transplantation for acute or subacute liver failure or who underwent retransplantation were excluded to avoid cohort heterogeneity from the PVT-RI development model. Patients who were known to have PVT at the time of listing were also excluded from statistical analysis. Patient characteristics analyzed included demographics (age at listing and transplantation, wait time from listing to OLT, gender, and ethnicity), etiology of liver disease, MELD score at listing, HCC, diabetes, and complications of portal hypertension, moderate-to-severe ascites, SBP, grade 3 or 4 hepatic encephalopathy, and insertion of TIPSS (Table 1).

**Table 1:** Patient demographics and baseline characteristics.

		**Incident PVT** **(*N =* 18)**	**No incident PVT** ** (*N =* 535)**	***p*-value**
Age, years				
	Mean	49.6	53.9	0.295
	Max	67	72	
	Min	23	19	
Gender (male)		10 (2.55%)	382 (97.45%)	0.145
Ethnicity				0.817
	European	14 (3.74%)	360 (96.26%)	
	Polynesian	2 (1.98%)	99 (98.02%)	
	Asian	2 (3.39%)	57 (96.61%)	
	Middle-eastern	0	8 (100%)	
	African-American	0	8 (100%)	
	Unknown	0	3 (100%)	
Wait time, days				
	Mean	228.7	131.2	**0.019**
	Max	532	1053	
	Min	2	0	
HCC (Yes)		1	234	**0.0013**
Etiology				
	Hepatitis C	4	178	
	Hepatitis B	1	111	
	Cholestatic/ Biliary pathology	6	90	
	NASH	0	43	
	Cryptogenic	1	12	
	Alcohol	3	51	
	Autoimmune	1	8	
	Others	2	42	
MELD score at listing	Mean	17.1	13.8	0.171
Diabetes (Y)		4 (4.08%)	94 (95.92%)	0.54
SBP (Y)		4 (4.3%)	89 (95.7%)	0.522
TIPPS (Y)		0	35 (100%)	0.62
Mod-severe		5 (4.85%)	98 (95.15%)	0.420
ascites				
HE > grade 2		0	15	1.00

This is bold to highlight statistically significant *p* Value.

Incident PVT was defined as a new diagnosis of PVT while on the waiting list (through imaging) or at the time of transplant (direct examination by the liver transplant surgeon or pathological examination of the explant). No incident PVT was defined as no evidence of PVT at listing and at transplantation.

### Statistical analysis

Initial statistical analysis was performed using the Mann Whitney test and paired *t*-tests. A biostatistician's help was sought to get further analysis and Kaplan-Meir survival curves.

## Results

A total of 706 adult patients received OLT between 1998 and February 2020. Out of these, 113 were excluded for acute/subacute liver failure or retransplantation, a further 40 patients who had known PVT at the time of listing were also excluded from statistical analysis. Data for analysis were collected for 553 patients who met the inclusion criteria. Of these, 18 (3.3%) patients were found to have incident PVT and 535 (96.7%) did not have incident PVT. The proposed PVT-RI calculator was unable to be validated in our cohort with only one patient scoring >4.6 which was proposed to have high accuracy in correctly predicting who would develop PVT post listing. A total of 11 patients were in the intermediate range (score 2.6–4.6) and six patients scored < 2.6 which was the proposed lowest cut-off. On the other hand, only 37.4% (200/535) of patients with no incident PVT scored < 2.6 which was proposed to rule out the incidence of PVT with high accuracy (negative predictive value 94%). There was no statistically significant difference between the mean and median values of PVT-RI scores of the two groups with a *p*-value of 0.245 (Table 2). The factors which were independent predictors for incident PVT were the absence of HCC and longer wait time for liver transplantation. One out of 18 (5.6%) patients with incident PVT had HCC whereas 234 out of 535 (43.7%) patients with no incident PVT had HCC. The mean wait time between listing and OLT was 228.7 days for the incident PVT group and 131.2 days for the non-PVT group. There was a statistically significant difference between the two groups with a *p*-value of 0.019, suggesting that a longer waiting time from listing to liver transplantation was associated with an increased risk of PVT development. There was no significant difference in the occurrence of incident PVT in viral vs. non-viral etiology or cholestatic vs. non-cholestatic chronic liver disease. In contrast to a few other international studies, our analysis did not show an increased incidence of PVT in patients with cirrhosis secondary to NASH.

**Table 2:** PVT-RI score comparison between patients with and without incident PVT.

		**Incident PVT** **(*N =* 18)**	**No incident PVT** ** (*N =* 535)**	***p*-value**
PVT-RI score	Mean	3.01	2.85	0.245
	Median	2.91	2.76	
No of pts with PVT-RI score (Column %)	< 2.6	6 (33.3%)	200 (37.4%)	
	2.6–4.6	11 (61.1%)	334 (62.4%)	0.086
	>4.6	1 (5.6%)	1 (0.2%)	

The mean age of recipients at OLT was 53.8 (± 10.3) years. The mean MELD score at listing was 13.9. The most common etiologies of underlying chronic liver disease were chronic hepatitis C (32.9%), chronic hepatitis B (20.3%), biliary cirrhosis (17.4%), alcohol (9.8%), and non-alcoholic fatty liver disease (7.8%). A total of 235 (42.5%) patients had Hepatocellular carcinoma.

**Figure 1:**
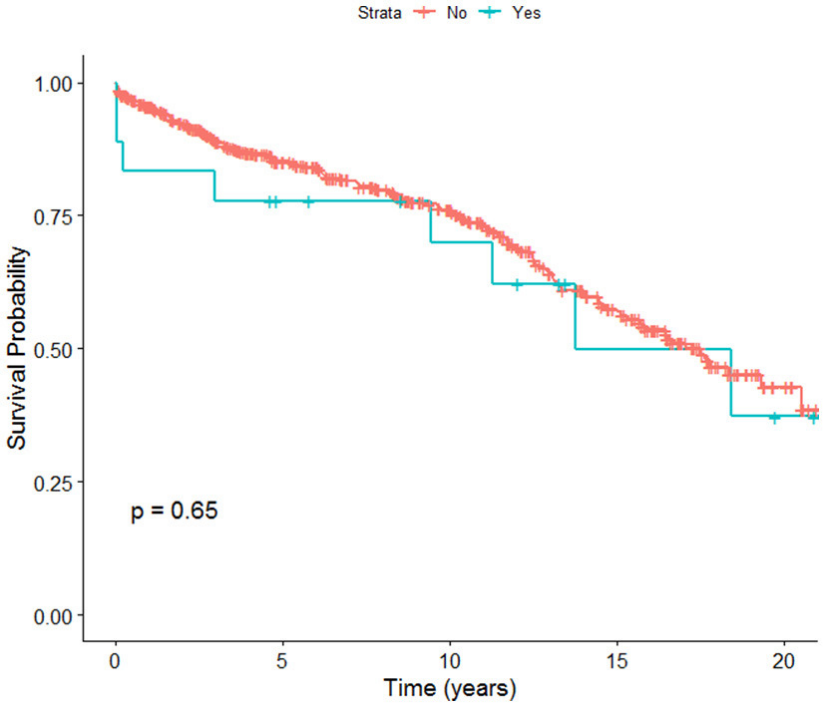
Overall survival of OLT recipients (incident PVT vs. no incident PVT).

Overall, post-transplant survival in adult OLT recipients in NZ was 95% at 1 year, 85% at 5 years, and 75.9% at 10 years. Early post-transplant survival rates were found to be higher in patients who did not have incident PVT compared to patients who were found to have incident PVT after being listed for post liver transplant. There was no significant difference in overall survival rates between patients with or without incident PVT at 20 years (Figure 1).

## Conclusion

The incidence of PVT in adult liver transplant recipients in this single center series was 3.3%. The proposed PVT-RI calculator did not prove to be useful in accurately predicting the incidence of PVT in NZ OLT recipients. Only one patient with incident PVT had a score of >4.6 which was proposed to have high accuracy in correctly predicting who would develop PVT post listing. Patients listed for liver failure were at higher risk of developing PVT compared to those listed for HCC.

Patients with longer waiting times on the transplant list should be closely monitored for PVT. We have promising overall survival rates post liver transplant of around 40% at 20 years in NZ.

## Discussion

Portal vein thrombosis is a well-recognized complication of chronic liver disease and cirrhosis. It is predictive of inferior outcomes both in the pretransplant and peri-transplant setting with increased rates of mortality and morbidity (6–9). This is contributed by multiple factors including slowing, stasis, and reversal of portal venous flow because of portal hypertension, a procoagulant environment in advanced liver disease caused by reduction of anticoagulant and anti-fibrinolytic proteins, such as anti-thrombin III, and specific systemic hypercoagulability associated with cholestatic liver disease. Ben-Ari et al. (19) performed thromboelastography to evaluate whole blood clotting and fibrinolysis in patients with cholestatic liver disease [Primary biliary cirrhosis (PBC) and Primary sclerosing cholangitis (PSC)], non-cholestatic liver disease, and healthy subjects (19). They showed hypercoagulability in patients with PBC and PSC in comparison to non-cholestatic liver disease and healthy subjects, irrespective of cirrhosis or bilirubin concentration. Identified risk factors for the development of PVT in transplantation candidates include the severity of liver disease, presence of hepatocellular carcinoma, and etiology of underlying liver disease (e.g., NASH and autoimmune hepatitis). There are no consensus guidelines for radiological surveillance for PVT on the waiting list and subsequent pretransplant management including TIPSS, or anticoagulation (10–14). A recent study reviewed multiple patient and disease characteristics and proposed a PVT-RI scoring system with careful evaluation of risk factors for incident PVT (18). The scoring model consisted of five clinical factors, namely MELD score at the listing, age, moderate-to-severe ascites, NASH as etiology of liver disease, and African-American race. It was found that patients with a low cut-off score of < 2.6 using this model were at reduced risk of development of PVT after listing (NPV 94%), whereas patients with a high cut-off score of >4.6 were at increased risk of incident PVT (PPV 85%).

In this current study, we aimed to validate the PVT-RI calculator in a large single-center series of all adult patients who have received liver transplantation in New Zealand since the beginning of the liver transplantation program in 1998 through February 2020. We identified factors associated with an increased risk of incident PVT in patients listed and waiting for a liver transplant. Transplant listing for liver failure rather than HCC and longer wait time from listing to transplant were independently associated with the incidence of PVT. There was no significant difference in the incidence of PVT with the etiology of liver disease, i.e., viral vs. non-viral or cholestatic vs. non-cholestatic liver disease. Patients with longer waiting times on the transplant waiting list should be closely monitored for PVT.

The PVT-RI calculator did not accurately predict the risk of incident PVT in New Zealand liver transplant recipients, possibly reflecting differences from the American population in patient characteristics and status at the time of listing for liver transplantation. We do acknowledge that out of the 18 patients who developed incident PVT, none had NASH as the primary etiology for chronic liver disease and none of them belonged to the African-American ethnic group, this certainly impacts the validity of the PVT-RI score in our cohort. Our cohort consists of a mixed population with 18.26% (101/553) Polynesian people and 10.67% (59/553) Asian people. Four (2.5%) of these patients developed incident PVT.

We have identified other potential reasons for contrasting findings in our study. The average waiting time between listing and liver transplantation in the original study was at least 365 days, whereas it was significantly shorter in our NZ cohort. Compared to the original study where the prediction model was devised, nearly twice as many patients were listed for HCC in this study, with a correspondingly lower average MELD score (14 vs. 17–19). Ongoing prospective studies might help to determine whether this can be a useful tool to help in triaging patients for liver transplantation listing. NASH was not found to be a statistically significant predictor of the incidence of PVT in our transplant recipients. Further studies are needed to continue monitoring this association in the NZ population, given the worsening obesity and fatty liver epidemics across the globe which will increase the demand for liver transplantation. NASH patients with PVT were found to be associated with inferior survival post liver transplant by Agbim et al. (9) The researchers found a 37% increased risk of graft failure and a 31% increased risk of overall death in patients with PVT compared to patients without PVT.

Patient demographic characteristics like age, gender, and ethnicity did not show any significant association with PVT development in transplant candidates. Although the liver transplant recipients in NZ belong to diverse ethnic groups with a different ethnic composition than North America, we do not believe this would have contributed to the differences in outcomes in this study compared with the original prediction model study. We did not find an association between the incidence of PVT with features of chronic and decompensated liver disease like MELD score, spontaneous bacterial peritonitis, moderate or severe degree of ascites, grade II or more hepatic encephalopathy, and TIPSS. A plausible explanation for this observation can be shorter waiting times between listing and transplantation in NZ as compared to North America.

Our study has some limitations. It is a single-center retrospective analysis. We acknowledge that it may be subject to the possibility of undetected confounding variables like other co-morbidities at the time of listing and the development of hepatic decompensation while on the transplant waiting list. On the other hand, this is the first study in the world to the best of our knowledge, to attempt to validate the PVT-RI model. We hope this leads to similar research in other transplantation centers which will enhance our knowledge and experience about potential predictive factors in the development of PVT in patients waiting for a liver transplant, its association with other patient and disease characteristics, and its impact on patient outcomes both pre- and post-transplant. Our sample size is relatively limited compared to the original study by Gaballa et al. (18). However, it includes every adult liver transplant recipient from the beginning of the national liver transplantation program in 1998 to February 2020. The overall rate of incident PVT development in our transplant recipients was only 3.3%. We have identified potential factors which may increase the risk of the development of PVT while on the transplant waiting list, which is a longer time on the waiting list and listing for liver failure rather than HCC. This study demonstrates 40% overall survival rates at 20 years post OLT, with relatively better survival rates in patients without incident PVT. Portal vein velocity has been found to be strongly associated with incidence of PVT (11, 20) and we did not have this information for our cohort.

In conclusion, the development of PVT prior to liver transplantation increases the waiting list and post-transplant mortality. Therefore, all patients listed for liver transplantation should undergo regular surveillance doppler USS with the initiation of anticoagulation if mesenteric venous thrombosis is identified. The development of a validated predictive model for PVT should allow earlier diagnosis and management such as anticoagulation and TIPSS, thereby improving pre- and post-transplant outcomes. Patients with longer waiting times for liver transplants should be closely monitored for the development of incident PVT. Further studies are needed to validate these observations.

## Data availability statement

The original contributions presented in the study are included in the article/supplementary material, further inquiries can be directed to the corresponding author.

## Ethics statement

Ethical review and approval was not required for the study on human participants in accordance with the local legislation and institutional requirements. Written informed consent for participation was not required for this study in accordance with the national legislation and the institutional requirements.

## Author contributions

PG conducted audit, data collection, and initial statistical analysis. BH assisted with initial data collection from New Zealand liver transplantation database. EG supervised the study and helped with statistical analysis. All authors contributed to the article and approved the submitted version.

## Conflict of interest

The authors declare that the research was conducted in the absence of any commercial or financial relationships that could be construed as a potential conflict of interest.

## Publisher's note

All claims expressed in this article are solely those of the authors and do not necessarily represent those of their affiliated organizations, or those of the publisher, the editors and the reviewers. Any product that may be evaluated in this article, or claim that may be made by its manufacturer, is not guaranteed or endorsed by the publisher.

## Abbreviations

HCC: Hepatocellular carcinoma
NASH: Non-alcoholic steatohepatitis
NPV: Negative predictive value
NZLTU: New Zealand Liver Transplant Unit
NZ: New Zealand
OLT: Orthotopic Liver Transplant
PBC: Primary biliary cirrhosis
PPV: Positive predictive value
PSC: Primary sclerosing cholangitis
PVT: Portal vein thrombosis
PVT-RI: Portal vein thrombosis risk index
SBP: Spontaneous bacterial peritonitis
TIPSS: Transjugular Intrahepatic Portosystemic shunt.
